# Evaluating attachment-based family therapy in residential treatment in the United States: does adolescents’ increased attachment security to caregivers lead to decreases in depressive symptoms?

**DOI:** 10.1186/s13034-024-00833-w

**Published:** 2024-11-13

**Authors:** Guy Diamond, Alannah Shelby Rivers, Payne Winston-Lindeboom, Jody Russon, Michael Roeske

**Affiliations:** 1 ABFT International Training Institute, Philadelphia, PA USA; 2 University of Pennsylvania, Perelman School of Medicine, Philadelphia, PA USA; 3 Texas Woman’s University, Denton, TX USA; 4 Newport Healthcare (Center for Research and Innovation), Nashville, TN USA; 5 Virginia Tech, 840 University City Blvd, Suite 1, Blacksburg, VA 24060 USA

**Keywords:** Attachment-based family therapy, Depression, Attachment, Adolescents, Residential treatment, United States

## Abstract

**Background:**

The inclusion of family therapy in residential treatment centers (RTCs) has increased over time. However, there is little data on whether empirically-supported family therapies (ESFTs) are being adopted and if they contribute to treatment effectiveness. This study aimed to test whether Attachment-Based Family Therapy (ABFT), an ESFT integrated into a large residential psychiatric system, would improve perceived attachment insecurity (anxiety and avoidance) and contribute to decreases in depression for adolescents.

**Method:**

ABFT was integrated into the clinical program of a large, residential psychiatric system. All family therapists were trained to a level of certification. Improvement was measured by changes in adolescent’s perceived attachment to caregivers and reduction in depressive symptoms. The sample included 4786 patients. Attachment insecurity and depressive symptoms were measured at intake, week 3, and week 5. A random-intercept, cross-lagged panel model was used to examine the relationships between attachment and depression over time.

**Results:**

The results generally supported hypotheses. Attachment insecurity and depressive symptoms improved over the five weeks of treatment. Improvements in attachment avoidance preceded improvements in depressive symptoms within subjects, over time. Simultaneously, improvements in depressive symptoms preceded those in both dimensions of attachment. Thus, improvement in perceived attachment was associated with a reduction in depressive symptoms.

**Conclusion:**

RTCs that can generate improvements in attachment insecurity and depressive symptoms, via ABFT or other ESFTs, might improve treatment outcomes, and ideally, adolescents’ successful transition back home to families. More research is needed to disentangle the contribution of ABFT and other treatment elements in a multimodal, residential treatment program. The study supports the call for increased incorporation of families into the RTC treatment process.

## Introduction

Psychiatric residential treatment centers (RTCs) serve an essential function in the continuum of mental health services for adolescents. A report from 2019 identified almost 600 RTCs for youth (up to age 18) in the United States [1]. These adolescent patients usually present as the most troubled in the mental health system, who have not benefited from less restrictive treatment settings or have struggles too difficult to address while living in a home environment [2]. The cost of these programs have been estimated to be between $23,000 and $55,000 a treatment episode [3] and many youth, with unsuccessful discharge statuses, require repeated residential placements [4]. Unfortunately, there is limited data on the effectiveness of RTC programs, minimal information on the adoption of empirically supported treatments in these settings, and little documentation on how RTCs incorporate family-centered care models. Addressing these challenges could improve outcomes in residential care and facilitate adolescents’ transition back home [5–7].

Family-centered care has been encouraged as a program philosophy to improve RTC services [5–7]. Numerous international policy statements and white papers have called for more family-centered care in these residential settings [8–10]. These papers recognize that a family-centered care framework can address the quality of family engagement and functioning, which, in turn, may impact youth mental health and the journey to recovery. Studies have consistently shown that negative family processes can cause or exacerbate mental health problems in youth [11, 12]. At the same time, positive and secure family relationships can protect against mental health distress [13]. In addition, even the highest functioning families can be destabilized when raising a child with mental health challenges and thus could benefit from additional support [14]. Several studies suggest that family involvement in RTC treatment leads to better treatment response, completion, and transition home [6, 10, 15–17]. Given that RTCs treat the most complicated patients and the cost of care is high, efforts should focus on understanding how to improve services and prevent subsequent residential placements. Family-centered care proposes that treating the entire family, not just the adolescent, may improve program outcomes and stability upon returning home [18–20].

One method for offering family-centered care is the incorporation of family therapy. This modality can reduce family risk factors (e.g., conflict, criticism) and increase family protective factors for psychosocial challenges. In line with family-centered care philosophy, improving how the family functions is a primary goal in addition to patient symptom reduction [21]. It is unclear from the literature, however, how often family therapy is delivered in RTC programs and which models have been implemented into these settings. Drawing on data from over 500 facilities, the 2018 National Mental Health Services Survey [22] found that family therapy was one of the least used modalities compared to individual, group and cognitive behavioral therapy (CBT). When family therapy is used, the details of the therapy approach are often not reported. In one recent review, Herbell and authors [23] questioned whether RTC programs used empirically supported family models and which family processes were targeted during treatment. Furthermore, family processes are rarely measured as a treatment outcome. When family outcomes are measured in these services, it is usually limited to frequency of contact or therapy attendance, and not family processes (e.g., attachment dynamics, warmth and structure, communication) that contribute to dysfunction [5, 6]. Therefore, little is known about which family therapy approaches may be effective in an RTC setting.

One candidate for family therapy in an RTC is Attachment-Based Family Therapy (ABFT) [24, 25]. This model has gained empirical support for the treatment of adolescents and young adults struggling with depression and suicidality mostly in outpatient studies [25]. The ABFT manual provides structure yet includes flexibility and guidance for clinical decision making across its five treatment phases or “tasks.” The model is trauma-informed, emotion-focused, and process-oriented. It addresses adolescent psychopathology through the mechanism of systemic family change. Several studies have demonstrated the effectiveness of ABFT for adolescents and young adults at risk for suicide [26–28]. Preliminary research also suggests ABFT may be effective for the treatment of youth struggling with eating disorders [29, 30] and anxiety [31, 32] as well as for younger children (ages 8 to 12) with a range of internalizing and externalizing distress [33]. Other studies have demonstrated ABFT can reduce depression and suicide ideation for LGBQ [34, 35] and trans-identified [36, 37] youth. In addition, ABFT has increased parental acceptance, decreased rejection, and improved adolescents’ attachment avoidance amongst sexual and gender minority young adults with non-accepting parents [38]. ABFT has been implemented in clinical settings including hospital-based psychiatric departments [39], child welfare systems [40], eating disorder programs [30, 41], LGBTQ + specialized services [37], and crisis units [42]. This extensive body of research has earned ABFT recognition as an empirically supported treatment for youth suicide and depression [43].

The ability of ABFT to work trans-diagnostically makes it well suited for implementation in RTCs, which treats a wide range of patients struggling with diverse and comorbid conditions. Training staff in multiple family therapy treatment modalities that target the range of disorders presented in these settings might not be feasible. Consequently, these clinical environments need flexible treatments that can be easily adapted for different clinical presentations. Although ABFT has been implemented in residential environments, little research has been conducted to demonstrate its effectiveness in these settings. One study in Australia provided initial evidence of the value of ABFT in a short-term crisis inpatient unit [42], but no other evaluation studies have been published. Given the potential of this intervention in these higher levels of care settings, more research is warranted.

### Proposed mechanisms of change in ABFT

ABFT proposes that, for many troubled youth, family and social stress drives a significant portion of psychiatric disturbance. While biological and temperamental vulnerabilities are relevant, the family environment may either buffer against psychopathology or exacerbate it [44]. Negative family environments (e.g., divorce, domestic violence, chronic criticism) or a family’s failure to adequately respond to stressful external events (e.g., bullying, sexual assault, discrimination) can rupture family relationships, which can further fuel psychiatric challenges. Many therapies aim to teach coping skills to better manage this distress. Others aim to identify and better understand how relational ruptures impact psychological health. ABFT uses both strategies to intervene in the family system. The model also brings these interpersonal disappointments into therapeutic conversations, so families can “work through” these experiences together. In doing so, families not only work to resolve family conflicts, but learn to communicate in healthier ways and gain confidence to successfully talk about difficult emotional experiences moving forward [25].

ABFT is grounded in attachment theory, which helps therapists understand the proposed mechanism of change. When children experience their caregivers as emotionally sensitive and available, they feel loved and protected, and they view the world as a trustworthy place where their needs can be met [45]. Children who grow up in emotionally responsive family environments develop a more secure sense of themselves and others compared to those who do not feel protected or attended to in their families. Unfortunately, some caregivers struggle to provide attachment-promoting parenting, as they may be impacted by current stressors, their own psychopathology, and, often, an unresolved history of their own attachment disappointments [46].

Under these conditions, children develop insecure attachment styles to protect themselves from getting emotionally hurt again. Specifically, a large body of research focuses on two common strategies, which may occur together or separately: attachment avoidance, and attachment anxiety [47, 48]. Youth with attachment avoidance protect themselves from further disappointment by rejecting or not expecting to be loved or supported by important others. Youth with attachment anxiety long to feel loved, but have low self-esteem, fear of abandonment, and mistrust of important others. Both of these insecure attachment strategies are frequently associated with depression [11, 49]. Because relational experiences shape attachment strategies, improvement in current relationship experiences can change one’s attachment style [44]. A central tenet in Bowlby’s framework is that internal working models are up for revision if parenting and relational environments can be improved (e.g., parent training, psychotherapy) [45]. In both adolescent and adult studies, improvements in attachment may be related to decreases in depression [50–52]. Consequently, many attachment-focused therapies aim to improve family processes to promote attachment security [53].

### The current study

The primary aim of this paper is to examine whether improvement in perceived attachment security, the primary target of ABFT, contributes to a reduction in depressive symptoms for adolescents in RTC services. We use data from a large, multistate, psychiatric residential treatment system that has adopted the ABFT model universally. All family therapists in the system are trained in ABFT and all patients receive this modality as a core component of treatment once or twice a week (depending on treatment phase). Depression and perceived attachment to caregivers are measured at program intake, week 3, and every two weeks until discharge. We hypothesized that improved perceived attachment security over time would be associated with subsequent reductions in depression. However, we recognized that there may be bidirectional associations between changes in depression and changes in perceived attachment over the course of treatment. Therefore, we used a random-intercept, cross-lagged panel model (RI-CLPM) [54] to examine these relationships over time.

## Methods

### Participants and procedure

The psychiatric system used for the study operates in multiple states, located in both rural and suburban environments. The facilities can house between six (house model) to fifty-three patients (campus model). The average age is around 15 with a mean length of stay of approximately 50 days. There is no use of restraints or locked rooms, with the exception of locking bathrooms following meals at certain locations treating disordered eating. However, staff are trained to engage in nonviolent crisis intervention de-escalation strategies that may include physical holds. Exclusion criteria for treatment in this system include active suicidal plan and intent with means that could be carried out in an RTC, IQ less than 70, history of assaultive behavior with police involvement, fire setting, cruelty to animals, or sexual perpetration behaviors, as well as disorientation or questionable competency. In addition to ABFT, this organization also utilizes Eye Movement Desensitization and Reprocessing (EMDR), Acceptance and Commitment Therapy (ACT), and Dialectical Behavioral Therapy (DBT) informed techniques, as well as psychiatric services and various experiential therapies, such as equine, yoga, art, and movement. The treatment teams are composed of a family therapist, individual therapist, counselor, psychiatric provider, and dietary consultant.

The sample included 4786 adolescents admitted into a comprehensive residential psychiatric treatment system for adolescents and young adults with significant mental health concerns. Demographics are shown in Table 1. Adolescents with missing length of stay in the residential system (*n* = 399) were excluded from the analyses and the totals above.

**Table 1:** Means and standard deviations

Variable	M (SD)
Age	15.47 (1.50)
Number of diagnoses	3.97 (1.61)
Length of stay	51.05 (28.63)
Variable	*n* (%)
Gender	
Female	2,442 (51.0%)
Male	1,764 (36.9%)
Non-binary	174 (3.6%)
Prefer not to answer	34 (0.7%)
Missing due to survey administration error	372 (7.8%)
Race	
Asian	19 (0.4%)
Black/African American	28 (0.6%)
Two or more races	79 (1.7%)
White	4,592 (95.9%)
Other	10 (0.2%)
Unknown	58 (1.2%)
Ethnicity	
Hispanic	6 (0.1%)
Not hispanic	4,753 (99.3%)
Unknown	27 (0.6%)
Primary diagnosis	
Major depressive disorder	3,262 (68.2%)
Generalized anxiety disorder	347 (7.3%)
Post-traumatic stress disorder	317 (6.6%)
Bipolar disorder	197 (4.1%)
Disruptive mood dysregulation disorder	102 (2.1%)
Unspecified mood affective disorder	101 (2.1%)
Attention deficit hyperactivity disorder	75 (1.6%)
Oppositional defiant disorder	56 (1.2%)
Obsessive compulsive disorder	33 (0.7%)
Cannabis use disorder	31 (0.6%)
Other	194 (4.1%)
Not available	72 (1.5%)

Data for the current study were collected from 2020 to 2022 as part of an outcomes monitoring program to examine treatment processes and results. At each patient’s intake meeting, an assessment battery was administered by staff through bhworks, an electronic platform designed to deliver self-report assessment tools for behavioral and mental healthcare organizations [55]. The full assessment takes approximately 15 min. The cadence of the follow-up assessments was determined by the outcomes monitoring program. Follow-up assessments were administered starting around week 3, so that clinicians would have the data before the first treatment plan review. Measures were repeated approximately every 2 weeks to evaluate progress and account for patients leaving the program. The first three assessments (intake, week 3, and week 5) were used for the current study, due to increasing amounts of missing data in part due to discharge after week 5. For participants with multiple admissions, only data from the first stay in the target period was used. The current study was deemed “exempt from IRB oversight” by the Advarra Institutional Review Board and was approved by the treatment center’s internal Research Review Panel.

### Standardizing ABFT

In 2018, this residential psychiatric system adopted ABFT as its core family therapy treatment modality. Prior to this, the agency offered an eclectic approach to family treatment and subsequently welcomed a standardized and empirically supported ABFT manual to train all therapeutic staff. The agency contracted with the ABFT International Training Institute [43] to provide ongoing training as new staff joined the organization. The training program involves multiple components. To begin, all clinical staff attend a one-day introductory course on ABFT (Level I certification). Family therapists then complete two more days of introductory training and participate in a year of biweekly supervision. In addition to their year of supervision, family therapists also complete an advanced three-day training. During supervision meetings, therapists present at least four times and submit a written ABFT-informed case presentation. Staff must also show video recordings of their work at least twice during the supervision period. At the end of this training sequence, staff have to pass a written test in order to achieve Level II certification. Selected family therapists are then invited to an advanced supervision group leading to Level III certification. Many of these Level III certified staff become clinical directors within the organization and one staff member became an internal ABFT trainer for the psychiatric system. During the three years of data collection for this study, the residential psychiatric system trained and certified approximately 150 therapists each year. Many of these training procedures mirror those used in clinical trials research, and training programs of other empirically-supported treatment models [25]. 

### Measures

#### Attachment

The Experiences in Close Relationships-Relationship Structures (ECR-RS) questionnaire [56] was used to assess attachment anxiety and avoidance involving parental figures. Previous research suggests the ECR-RS has good internal consistency and factor validity [56]. Adolescents were asked to complete the scale for two parental figures, at each of the three time points assessed in the present study (intake, week 3, and week 5). Adolescents indicated their relationship with attachment figures in a free response format, but this information was removed as part of the organization’s de-identification process before these analyses were conducted. This question was later converted to a drop-down selection (e.g., father, mother, grandmother) which provided additional information while preserving de-identification efforts. Only a small subsample of adolescents, however, received this form of the question. Because the relationship with the attachment figure was unknown for most of the sample, and scores were highly correlated across figures, attachment scores were averaged across both figures to create overall indices of anxiety and avoidance (as recommended for this measure) [57]. Cronbach’s alphas were 0.87 (anxiety) and 0.90 (avoidance) at intake, and similar at subsequent time points.

#### Depressive symptoms

The Patient Health Questionnaire-9 (PHQ-9) [58] was used to assess depressive symptoms. The PHQ-9 is brief, with strong sensitivity and specificity [59]. Cronbach’s alpha was 0.90 at intake, and similar at subsequent time points.

### Approach to analysis

**Fig. 1:**
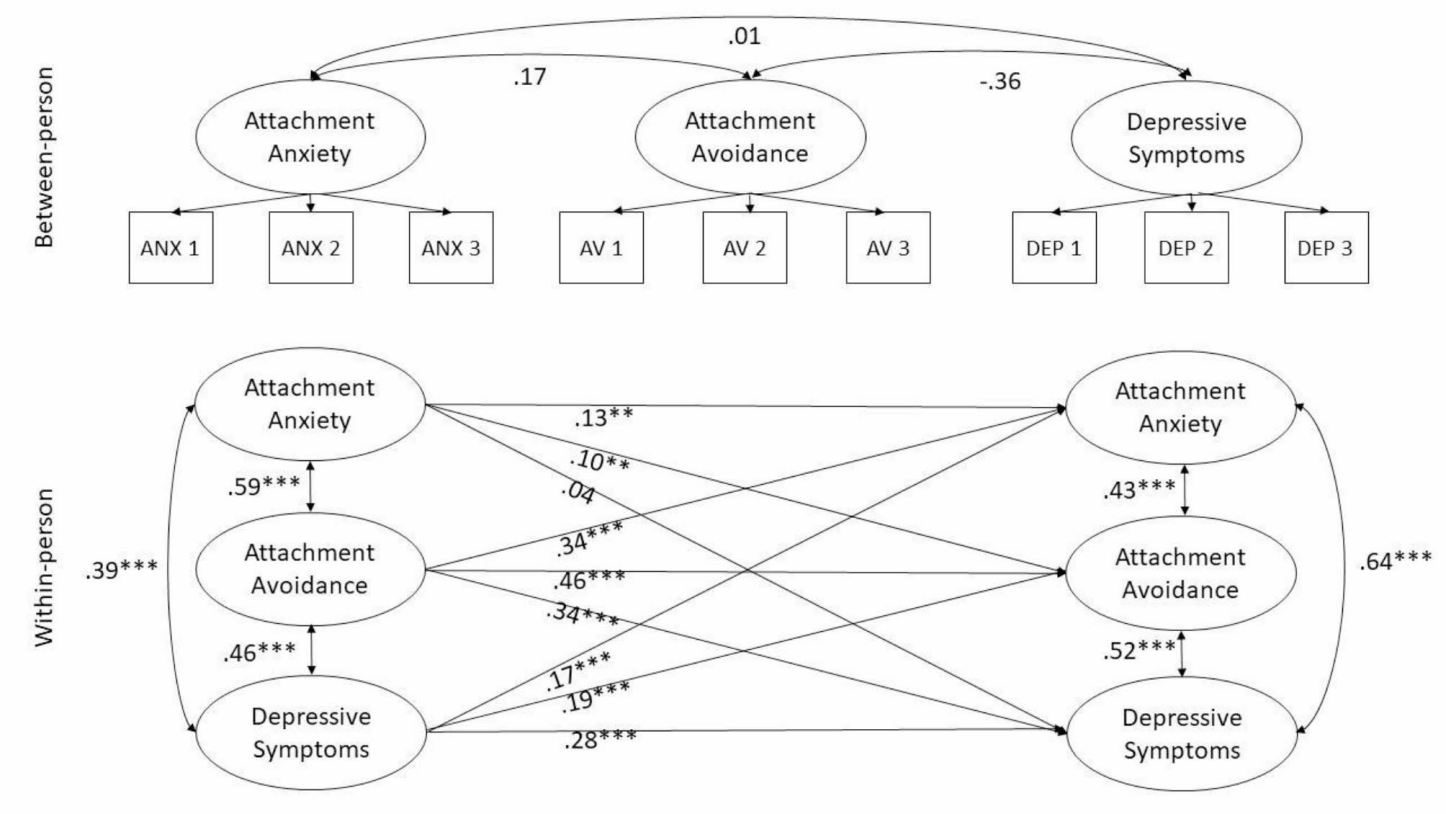
Random intercept cross-lagged panel model. Length of stay was included as a time-invariant control

A random-intercept, cross-lagged panel model (RI-CLPM) [54] was estimated to examine relationships over time. The model is shown in Fig. 1. The RI-CLPM models associations at both the between-person (stable individual differences) and within-person (change over time) levels, which allows for more accurate estimates compared to a traditional cross-lagged panel model. The model was specified with three time-varying variables: attachment avoidance, attachment anxiety, and depressive symptoms. Length of stay was also included as a time-invariant control variable (not shown in the figure). Missing data were handled with full-information maximum likelihood. The initial model constrained estimates (means, lagged and autoregressive effects, residual variances and covariances) to be equal over time. A well-fitting model was specified a priori as CFI ≥ 0.95, SRMR ≤ 0.08, and RMSEA ≤ 0.06 (Hu & Bentler, 1999).

## Results

The initial model, constraining all estimates to be equal across time, fit poorly (χ^2^(28) = 726.63, *p* < 0.001; CFI = 0.945, RMSEA = 0.072, SRMR = 0.103). See Table 2 for the means. This was not surprising given mean change over time was expected, and examination of the modification indices indicated constraining the means over time was problematic. Therefore, the between-person components represent how much an individual adolescent’s trajectory is above or below the mean trajectory (while sharing the same underlying trend of improvement), and the within-person components as increases and decreases relevant to that adolescent’s trajectory [60]. Upon freeing the means to vary, the model fit well; χ^2^(24) = 179.12, *p* < 0.001; CFI =0.988, RMSEA = 0.037, SRMR = 0.027. Therefore, this model was used for subsequent analyses. Notably, this implies that relationships from baseline to week 3, and week 3 to week 5, could be constrained to be equal, and therefore did not significantly differ.

**Table 2:** Means and standard deviations

Variable	Intake	Week 3	Week 5
Attachment anxiety	2.79 (1.55)	2.64 (1.58)	2.43 (1.56)
Attachment avoidance	4.11 (1.41)	3.70 (1.52)	3.41 (1.55)
Depressive symptoms	12.44 (7.78)	8.14 (7.20)	7.13 (6.87)

Figure 1 shows the primary results. On the between-person level (top), there were no significant associations; that is, after accounting for variations within individuals over time, average levels of attachment insecurity and depressive symptoms were not associated across individuals. On the within-person level (bottom), there were significant positive autoregressive effects; higher within-person centered scores at one point predicted higher relative scores at the next time point for all three variables. There were positive lagged associations for all pairs of variables except for attachment anxiety predicting later depressive symptoms. That is, having elevated attachment avoidance or depressive symptoms at one time predicted elevated attachment anxiety, avoidance, and depressive symptoms at the subsequent time. Finally, there were positive cross-sectional (within time point) associations between all three variables, both at time one (unconditional associations) as well as later time points (conditional associations after accounting for lagged and autoregressive effects).

## Discussion

This study aimed to test one of the key theoretical tenets of ABFT; namely, that improving adolescents’ perceived attachment to caregivers should lead to reduction in depressive symptoms. This study was unique in testing these changes in the context of an RTC setting. Across three time points in a random intercept cross-lagged panel model, the results generally supported our hypotheses. Attachment insecurity and depressive symptoms improved over 5 weeks of treatment and, within the same adolescents over time, improvements in attachment avoidance preceded improvements in depressive symptoms. At the same time, improvements in depressive symptoms preceded improvements in both dimensions of attachment (anxiety and avoidance). After accounting for these cross-lagged associations, cross-sectional associations remained within adolescents. That is, at times when adolescents had elevated attachment insecurity (after accounting for individual differences) they were also likely to report greater depressive symptoms. However, no between-person associations were significant. This means that, after accounting for within-person associations over time, those with a higher mean level of attachment insecurity did not also have a higher mean level of depressive symptoms. Taken together, change in attachment avoidance appears to be a plausible mechanism for some, but not all, of the change in depressive symptoms. The relationship between changes in attachment insecurity and depressive symptoms is bidirectional. These findings offer some support for the theoretical mechanisms of ABFT, as well as attachment-informed, family-focused residential treatment programs.

Little research has examined how change in attachment and depressive symptoms influence each other over time. Some evidence suggests, however, that improvements in attachment may be related to decreases in depression for adolescents and adults in treatment [50–52]. Based on the results of the present study, it is worth considering why only reductions in attachment avoidance, and not attachment anxiety, predicted subsequent improvements in depressive symptoms. While the literature on attachment dimensions as outcome moderators or mediators is limited and somewhat mixed, child and adult patients with attachment anxiety tend to benefit less from psychotherapy across a wide range of treatment modalities [61]. This might be explained by these patients’ approach-avoidance orientation [62]. Specifically, patients with attachment anxiety present with a mix of bids for comfort and support from important others along with withdrawal and anger because they believe their efforts will fail. In the context of family treatments for youth, it may be more challenging to coach parents to interact with these adolescents in a way that can revise their internal working models, that is, help them begin to see their caregiver as capable of providing comfort. In contrast, for youth with attachment avoidance, helping parents learn and employ more steady, attachment-promoting behaviors is often obtained in ABFT [63]. Indeed, over the course of the treatment period, reductions in attachment anxiety scores were also less than those for attachment avoidance. Further research should examine these changes in more depth in order to understand what therapeutic, contextual, or individual factors influence unique changes in attachment anxiety and avoidance.

### Limitations and strengths

First, we recognize that drawing conclusions about the mechanisms of ABFT within a multimodal treatment program, like residential care, can be misleading, particularly without a control group. Patients are not only receiving other treatments (e.g., medication, individual, group, psychiatric, experiential therapies), but also simply being away from the home environment might change the way adolescents perceive their familial relationships. These potential confounds present some of the biggest challenges for outcomes research in this treatment setting, where the implementation of a control group might not be clinically feasible or ethical [64]. Therefore, many outcome studies of specific treatment modalities delivered in residential care deduce modality effectiveness from general, pre-post patient data and do not account for the impact of multiple treatments or the possible role of regression to the mean [65]. In our study, we focused on the measurement of a primary ABFT treatment target (i.e., perceived attachment insecurity) that might not have been addressed if ABFT was not part of the program design. Still, attributing our adolescent outcomes to ABFT must be interpreted cautiously, as more research on this question is needed. It is possible that the adolescents would have shown some improvement on attachment and/or depressive symptoms without treatment (e.g., regression to the mean), or that other treatments (e.g., medication, ACT) could play a role. Information on specific treatment packages, including medication, received for each individual adolescent is not available; all patients receive multiple treatments. Future studies could explore whether outcomes vary due to the number of family sessions used to complete each task of ABFT treatment. Alternatively, future research could compare ABFT treatment as usual with an enhanced version, whereby some patients are given additional family therapy sessions.

Second, the sample is predominantly White and Non-Hispanic due to the private insurance base and demographics of the clinical population that come to the program, and results may not generalize to other racial groups. Relatedly, most youth in the sample had a primary diagnosis of a mood disorder, so the results might not generalize to adolescents with other diagnoses. This is especially worth noting for personality disorders, which were rarely included as a primary diagnosis for adolescents in this sample. This may be in part because the program is located in the United States and uses the Diagnostic and Statistical Manual-5 rather than the International Classification of Diseases-11, and the more categorical framework of the former appears to lead to lower rates of diagnosing personality disorders in adolescence [66, 67]. Third, implementation of ABFT occurred prior to the development of the outcomes monitoring program, from which the clinical data was collected. Therefore, there is no ability to compare changes in attachment insecurity and depressive symptoms without use of the empirically supported family therapy. Fourth, we recognize that adoption of an empirically supported treatment, like ABFT, in a large clinical facility, requires organizational commitment and resources. Replication of this study and a closer examination of the implementation procedures and barriers in this psychiatric system might help elucidate some of the findings.

While some methodological limitations exist, the study has several strengths. First, it includes a large sample with patients from over 40 clinical sites across the United States. Second, although no formal fidelity checklist was used to validate the model, the organization put into practice policies and procedures that ensured adherence (e.g., training, year of supervision, posttests). Third, most of the research on family factors in residential care uses static data (i.e., electronic medical record [EMR] data with frequency of family contacts or visits) to assess family involvement [5, 6]. In this paper, we have examined a core target self-report mechanism of the treatment, that is, patient improved perceived attachment with their caregivers. Fourth, the RTCs implemented a well-known and psychometrically sound attachment insecurity measure [56] into the program evaluation assessment battery. This measure was collected several times over the course of treatment, thus allowing for the analytic modeling of the primary study questions. Using RI-CLPM allowed for modeling the theoretically anticipated bidirectional effects while accounting for between-person differences. This presents a more accurate picture of the rapid changes that can occur during residential treatment.

### Clinical implications

Though not the primary aim of this study, the paper provides evidence that an ESFT model can be adopted by a large RTC organization. By standardizing training procedures and program expectations, all family therapy staff were able to be trained to a level of acceptable fidelity to the model (e.g., certification). In addition, the incorporation of ABFT helped standardize the organization’s family-focused commitment and principles, provided structure to clinical procedures, and enhanced commitment to family therapy as a primary modality. The ABFT model specifically offers a strategy to place attachment repair at the center of clinical conversations, thus increasing the likelihood that all patients will receive attention to this common clinical challenge.

In terms of demonstrating ABFT’s mechanism of change, the study suggested that a change in perceived attachment avoidance to caregivers could lead to a reduction in depressive symptoms. This is consistent with a large body of research linking family functioning to depressive symptoms [68]. In addition, a core assumption of attachment theory is that, by increasing a secure base, attachment to caregivers can increase the adolescent reliance on caregivers for support and problem solving [61]. Thus, RTC programs that can accomplish this clinical goal (using ABFT or other forms of therapy) might potentiate clinical outcomes and stability when the patient returns home.

## Conclusion

Measuring the contribution of a single modality in a multimodal RTC can be challenging. This study aimed to examine and measure the core proposed change mechanisms of a family therapy model and of the program: improving patients’ perceived attachment to caregivers. Extensive literature links insecure attachment to depression as well as to a host of psychological and interpersonal skills (e.g., emotional regulation, perspective taking, self-awareness, ability to engage in therapy) [11, 61, 69]. While there are many therapy targets for youth placed in an RTC, investing in improving attachment security presents a robust clinical goal that may potentiate treatment outcomes. Compared to an individual therapy format that may have similar clinical aims, ABFT may allow therapists to not only help the youth, but also to potentially help parents by improving the parenting behaviors that will contribute to a secure base and improved emotional life when the child returns home. In addition, the model appears to be adaptable and well-suited to an RTC. This effort to strengthen youth and parents’ skills and relationships is consistent with the family-centered care model encouraged for RTCs [8–10] and shows promise as a pathway toward improved clinical outcomes and family relationships.

## Data Availability

The data are not available; the participants of this study did not give written consent for their data to be shared publicly, so due to the sensitive nature of the research supporting data is not available.
